# Transcriptome and metabolome analyses provide insights into the relevance of pericarp thickness variations in *Camellia drupifera* and *Camellia oleifera*

**DOI:** 10.3389/fpls.2022.1016475

**Published:** 2022-10-27

**Authors:** Yongjuan Li, Boyong Liao, Yi Wang, Huihua Luo, Shimin Wang, Caiqin Li, Wenpei Song, Kunchang Zhang, Boqun Yang, Shaoqiang Lu, Bipei Zhang, Yongquan Li

**Affiliations:** ^1^ College of Horticulture and Landscape Architecture, Zhongkai University of Agriculture and Engineering, Guangzhou, China; ^2^ State-owned Xiaokeng Forest Farm in Qujiang District of Shaoguan City, Shaoguan, China

**Keywords:** *Camellia drupifera*, *Camellia oleifera*, pericarp thickness, lignin, transcriptome, metabolome

## Abstract

*Camellia* fruit is a woody edible oil source with a recalcitrant pericarp, which increases processing costs. However, the relevance of pericarp thickness variations in *Camellia* species remains unclear. Therefore, this study aimed to identify pericarp differences at the metabolic and transcription levels between thick-pericarp *Camellia drupifera* BG and thin-pericarp *Camellia oleifera* SG. Forty differentially accumulated metabolites were screened through non-targeted UHPLC-Q-TOF MS-based metabolite profiling. S-lignin was prominently upregulated in BG compared with SG, contributing to the thick pericarp of BG. KEGG enrichment and coexpression network analysis showed 29 differentially expressed genes associated with the lignin biosynthetic pathway, including 21 genes encoding catalysts and 8 encoding transcription factors. Nine upregulated genes encoding catalysts potentially led to S-lignin accumulation in BG pericarp, and transcription factors NAC and MYB were possibly involved in major transcriptional regulatory mechanisms. Conventional growth-related factors WRKYs and AP2/ERFs were positively associated while pathogenesis-related proteins MLP328 and NCS2 were negatively associated with S-lignin content. Thus, *Camellia* balances growth and defense possibly by altering lignin biosynthesis. The results of this study may guide the genetic modifications of *C. drupifera* to optimize its growth–defense balance and improve seed accessibility.

## Introduction

*Camellia drupifera*, a newly identified *Camellia* species serving as a woody edible oil crop similar to oil palm, olive, and coconut (Long et al., 2008; Wang et al., 2014; Chen et al., 2015), is grown specifically in South China and genetically proximal to *Camellia oleifera* (Flora of China, 1994; Qin et al., 2018). Oil extracted from *Camellia* seeds is rich in monounsaturated fatty acids and other bioactive metabolites with anticancer, antioxidant, and immunity-enhancing effects, thereby becoming a healthy high-grade edible oil in the global market these days (Suealek et al., 2021; Liu et al., 2022). Increased attention has been drawn to cost-efficient oil production from seeds that are embedded in dry fruits or capsules. The capsule of *Camellia* is anatomically composed of seeds, pericarp, and carpel bundles. Dry dehiscent capsules are split *via* three to five valves, with each section holding one to four seeds when the fruit is ripe (Patrick, 1997; Ming, 2007; Orel and Wilson, 2012).

Oil-rich seeds are under firm protection of pericarps against external abiotic and biotic stresses before maturation; however, the recalcitrance of woody pericarps (a matrix of lignocellulosic materials) increases processing costs for seed accessibility (Li et al., 2016). Therefore, pericarp thickness has been considered an economic structural trait of *Camellia* species, where thin ones are favored over thick ones.

Tan et al. (2020) identified the constituents of woody pericarps in *C. oleifera* Abel to be 15.8% cellulose, 23.6% hemicellulose, 8.8% lignin, and others, which include polymers deposited in secondary cell walls. Lignin polymers cross-link with cellulose microfibrils and hemicellulose molecules *via* side chains, forming a rigid cell skeleton and rendering the pericarp recalcitrant (Reddy et al., 2005; Chen and Dixon, 2007; Henry, 2010).

Different amounts and/or compositions of lignocellulosic constituents produce distinct pericarp thicknesses in *Camellia* species. However, the relevance of pericarp thickness variations in *Camellia* species and the underlying regulatory mechanisms remain unclear, complicating the genetic modification of this trait. Genes involved in the biosynthesis of some components, particularly cellulose and lignin, have been characterized in other plants.

The pericarp of *C. drupifera* capsules is notably thicker than that of *C. oleifera*. Both species are endemic to South China, providing ideal materials for investigating the relevance of pericarp thickness variations in *Camellia* species. Apart from pericarp thickness, some coupled properties related to growth and defense have also aroused wide concern. For instance, thick pericarps are usually accompanied by tall trunks and large capsules but weak defense, whereas trees with thin pericarps always exhibit strong defense but dwarf trunks and small capsules (Yang et al., 2015; Hong et al., 2016).

The present study aimed to compare the metabolic and transcriptional profiles of capsules from thick-pericarp *C. drupifera* BG (hereafter BG) and thin-pericarp *C. oleifera* SG (hereafter SG) and elucidate the mechanism by which pericarp thickness influences growth–defense tradeoffs in *Camellia*. In the current study, we found that syringyl lignin (S-lignin) was significantly upregulated in BG compared to SG, resulting in a thicker peel in BG. These results provide insights into the molecular basis of pericarp thickening in *Camellia* species. This study may guide the genetic modifications of *C. drupifera* to optimize its growth–defense balance and facilitate seed accessibility.

## Materials and methods

### Plant materials and sample preparation

Thick-pericarp *C. drupifera* BG and thin-pericarp *C. oleifera* SG were planted in the Boluo Forest Farm and the Xiaokeng Forest Farm Guangdong Province, China, under the same growth conditions. Fruit samples from individual trees were collected randomly from different branches in November 2020. Fresh capsules were dissected using a blade on ice bed, and their pericarps were separated manually while wearing sterile gloves, frozen immediately in liquid nitrogen, and then stored at -80°C for RNA extraction and metabolite analysis. Three biological replicates of each sample were used for RNA sequencing, and eight biological replicates were used for metabolic profiling. In the present study, the pericarp samples of the BG and SG capsules were abbreviated as BG_1, BG_2, BG_3… SG_1, SG_2, SG_3…

### Capsule phenotypes between BG and SG

The transverse, longitudinal, and horizontal diameters and pericarp thickness of 25 capsules were measured with a vernier caliper, and the fresh weight of seeds, fresh weight of pericarps, and weight of a single capsule were determined. The pulp at the transverse diameter was selected for the determination of pericarp thickness. A comparative analysis of the capsule phenotypic characteristics of BG and SG was performed using Excel software.

### Cellulose, hemicellulose, and lignin contents between two species of *Camellia* L.

The cellulose and hemicellulose contents of 0.5 g samples were prepared and quantified as previously described by Yan et al. (Yan, 2020). Lignin was assayed by derivatization with acetyl-bromide-glacial acetic acid (Barnes and Anderson, 2017). The pericarp was ground and filtered through a 60-mesh screen, and 5 mg of pericarp powder was added to 10 mL of 10% acetyl bromide–glacial acetic acid solution. and then, 500 μL of 70% perchloric acid was added and heated at 50°C for 30 min. After cooling, the reaction was terminated by mixing 10 mL of 2 mol/L NaOH and 10 mL of glacial acetic acid. Subsequently, the samples were centrifuged again at 8000 × r for 5 min. Absorbance was subsequently obtained at 280 nm using glacial acetic acid as a control.

### Toluidine blue reagent staining

For lignin characterization, the pericarps were stained with toluidine blue O (TBO) reagent (Soleibo Technology Co., Ltd., Beijing, China) as described previously (Jia et al., 2015). Pericarp specimens were cut from the middle part of the capsules and then soaked overnight in 70% formaldehyde alcohol acetic acid fixative solution. Paraffin sections were immediately stained with 0.05% (w/v) TBO for 10 min. The specimens were washed, dehydrated with an ethanol series of 75%–95%, and then embedded in neutral resin. The samples were sectioned using an ultrathin semiautomatic microtome (Lerca-RM2235, Germany) to prepare paraffin sections in accordance with the manufacturer’s instructions. Micrographs were taken under a Leica microscope (DM2000 LED, Germany).

### Untargeted metabolic analysis

Freeze-dried samples were crushed using the liquid-nitrogen grinding method. Powdered tissue (100 mg) was dissolved in 1.0 mL of cold methanol/acetyl cyanide (50:50 v/v) and blended twice by low-temperature ultrasonic treatment for 30 min. Extraction was stable at -20°C for 60 min, and then the homogenate was centrifuged at 14,000 × g for 20 min at 4°C. The supernatant was used for Liquid Chromatography-Mass spectrometry.

Metabolites were analyzed using an ultra-performance liquid chromatography system (UHPLC, Agilent 1290 Infinity LC system, USA) equipped with a HILIC column (1.7 μm, 2.1 mm× 100 mm column). The column temperature was maintained at 25°C, the flow rate was 0.3 mL/min, and the injection volume was 2 μL. Water with 0.04% ammonium acetate (v/v) and 0.04% ammonia and acetonitrile were used as the compositions of mobile phases A and B, respectively. Gradient elution was performed as follows: 0–1 min, 85% B (v:v); 1–12 min, 85%–65% (v:v); and 12–12.1 min, 65%–40% B (v:v), 12.1–15 min, 40%–85%, 15.1–20 min, 85% B, with automatic injection at 4°C during the whole analysis.

Primary and secondary spectra of the samples were collected using an AB Triple TOF 6600 mass spectrometer (AB Sciex, Concord, Canada). The ESI source conditions after HILIC chromatographic separation were as follows: Ion Source Gas1, 60 psi; Ion Source Gas2, 60 psi; curtain gas, 60 psi; source temperature, 600°C; Ion Sapary Voltage Floating ±5500 V (positive and negative modes); TOF MS scan m/z range, 60–1000 Da; product ion scan m/z range, 25–1000 Da, TOF MS scan accumulation time, 0.20 s/spectra; product ion scan accumulation time, 0.05 s/spectra; and declustering potential (DP), ± 60 V (positive and negative modes).

Raw MS data (.wiff scan files) were converted to mzML files using ProteoWizard (Chambers et al., 2012). The peak alignment, retention time alignment, and peak area were processed using XCMS software. The following parameters were used for peak picking: centWave, m/z = 25 ppm; peak width, c (10, 60); and prefilter, c (10, 100). The following parameters were used for peak grouping: bw, 5; mzwid, 0.025; and minfrac, 0.5. Metabolite identification was based on an in-house database at Shanghai Applied Protein Technology Co. Ltd. (Shanghai, China) established with authentic standards. After normalization to total peak intensity, the processed data were uploaded into SIMCA-P14.1 (Umetrics, Umea, Sweden) for mode identification, where they were subjected to multivariate data analyses, including principal component analysis (PCA) and orthogonal partial least squares discriminant analysis (OPLS-DA). The variable importance in projection (VIP) scores of each variable within the OPLS-DA model were calculated to indicate their contribution to their classification.

### Transcriptome sequence processing and annotation

Total RNA was extracted from the BG and SG capsules by using TRIzol Reagent (Magen, Guangzhou, China) and purified using the AMPure XP system (Beckman, USA). Paired-end libraries were prepared using an ABclonal mRNA-seq Lib Prep Kit (Abclonal, China) following the manufacturer’s instructions. Sequencing was performed using an Illumina NovaSeq 6000 instrument (Shanghai Applied Protein Technology Co. Ltd., Shanghai, China).

Raw data in fastq format were processed using Perl scripts. Clean reads were obtained by removing adapter sequences, low-quality reads (number of lines with a string quality value less than or equal to 25 accounts for more than 60% of the entire reading), and reads with N ratio (base information cannot be determined) greater than 5%. High-quality reads were assembled into contigs, transcripts, and unigenes by using Trinity software (http://trinityrnaseq.sourceforge.net/).

FeatureCounts (http://subread.sourceforge.net/) was used to count the number of reads mapped to each gene. Then, the FPKM of each gene was calculated based on the length of the gene and the read count mapped to this gene. Differential expression analysis was performed using DESeq2 (http://bioconductor.org/packages/release/bioc/html/DESeq2.html) (Love et al., 2014).

For functional annotation and classification, the assembled transcriptome sequences were compared to obtain annotation information in each of the five following databases: NR (http://ftp.ncbi.nlm.nih.gov/blast/db/) (Deng et al., 2006), Swiss-Prot (http://web.expasy.org/docs/swiss-prot.guideline) (Rolf et al., 2004), Pfam (http://pfam.xfam.org/) (Finn et al., 2014), gene ontology (GO, http://www.geneontology.org) (Ashburner et al., 2000), and Kyoto Encyclopedia of Genes and Genomes (KEGG, http://www.genome.jp/kegg/) (Minoru et al., 2016).

GO and KEGG enrichment analyses were conducted using the clusterProfiler R software package to explain the functional enrichment of DEGs and clarify the differences between samples at the gene function level. The GO or KEGG functions were significantly enriched when P < 0.05.

### Coexpression analysis of the lignin biosynthetic pathways

The reads count from RNA-seq reads and the sinapyl alcohol value from metabolome data were calculated correlation and p-value using the R platform (version 4.0.5). Then, lignin-related metabolic compounds and catalytic enzymes in Camellia and other plants were investigated from NR, Pfam, Swissprot, GO and KEGG databases and extracted from the calculated results. Co-expression network patterns were visualized by cytoscape (version 3.9.1, Shannon et al., 2003).

### Quantitative real-time polymerase chain reaction analysis of selected genes

Single-stranded cDNAs were synthesized from the RNAs using the PrimeScript™ RT reagent Kit, and quantitative real-time PCR was performed using ViiA7 (Thermo Fisher Scientific, USA) and Hieff™ qPCR SYBR Green Master Mix (Yisheng Biotechnology, Shanghai, China). Primers were designed by Bioruqi (Guangzhou, China) and synthesized by GENEWIZ (Suzhou, China). The glyceraldehyde-3-phosphate dehydrogenase gene (GAPDH) was used as an internal reference, and the relative expression was calculated using the 2ΔCt method. The standard errors of the means among the replicates were calculated. All quantitative real-time polymerase chain reaction (qRT-PCR) analyses were performed in three biological replications, respectively. The expression patterns of eight transcripts were monitored, and detailed information about the unigene IDs, fold change (FC), and primer pairs designed in this study are presented in **Supplementary Table S1**.

### Single-copy orthologous gene calling and phylogenetic analysis

Raw transcriptome data from BG and SG were uploaded to the SRA database (https://www.ncbi.nlm.nih.gov/sra). Other raw transcriptome data of five sibling *Camellia* species, including *C. japonica* (SRR17275277), *C. chekiangoleosa* (SRR15420647), *C. petelotii* (SRR17460028), *C. azalea* (SRR7120561), and *C. oleifera* (SRR17365493), were downloaded from the SRA database. Paired-end sequencing datasets were first trimmed with Trimmomatic (0.39) (http://www.usadellab.org/cms/?page=trimmomatic) using default settings, and then quality checks were performed using fastQC (0.11.9) (https://www.bioinformatics.babraham.ac.uk/projects/fastqc/) to confirm the removal of adapters and low-quality regions. The obtained sequences were then assembled using Trinity (2.9.1). Protein sequences were deduced using ORFfinder (https://ftp.ncbi.nlm.nih.gov/genomes/TOOLS/ORFfinder). All parameters were set to their default values. Single-gene orthologs were identified using OrthoFinder to cluster the protein sequences from the seven species (Emms and Kelly, 2019). Multiple sequence alignments were performed based on the amino acid sequences using the alignment tool MUSCLE with default parameter settings. Maximum-likelihood phylogenetic trees were constructed using MEGA7 software with the JTT model (Sudhir et al., 2016). In the phylogenetic tree, bootstrap supporting values below 50 were generally regarded as unreliable and are not shown. The 334 single-copy nuclear genes were conserved in eggNOG (http://eggnog5.embl.de/#/app/home) (Huerta-Cepas et al., 2019). The pictures of other *Camellia* species are from the plant plus of China (http://www.iplant.cn).

## Results

### Comparison of capsule phenotypes between BG and SG

**Figure 1:**
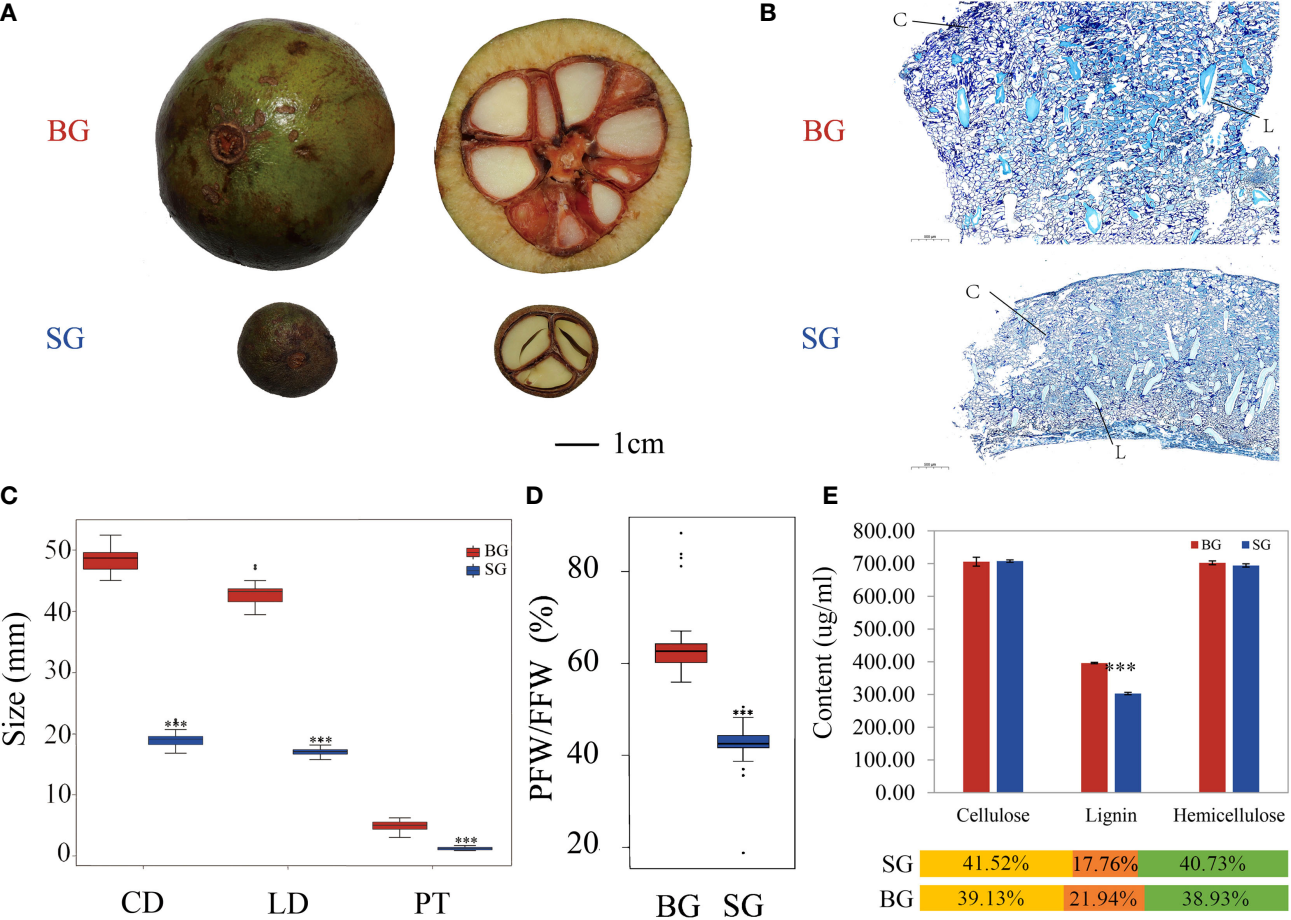
Comparison of fruit phenotypic characteristics between BG and SG. **(A)** Capsules of *C drupifera* (BG) and *C oleifera* Hu (SG); **(B)** Microscopic analyses of pericarps from BG and SG capsules; L, lignified cell wall; C, cellulose cell wall. **(C)** Comparison of cross diameter (CD), longitudinal diameter (LD), and pericarp thickness (PT) between BG and SG **(D)** Rate of pericarp fresh weight (PFW) to fruit fresh weight (FFW) between SG and BG. **(E)** Cellulose, lignin, and hemicellulose contents of BG and SG. Asterisks indicate a significant difference compared with BG; ***P < 0.001, Student’s t-test; Yellow: cellulose, orange: lignin, and green: hemicellulose.

We quantitatively characterized and compared the pericarp thicknesses of BG and SG capsules at mature developmental stages. As shown in **Figures 1A, B**, the BG capsules had a thicker pericarp than the SG capsules. Further quantification of capsule size in terms of cross and longitudinal diameters, pericarp thickness, and pericarp proportion supported the different capsule phenotypes observed between the BG and SG samples (**Figures 1C, D**). Moreover, lignin content, which contributes to mechanical support, was approximately 30% higher in the BG capsules than in the SG capsules. Although cellulose and hemicellulose were the main components of secondary cell walls, their contents did not significantly differ between the BG and SG capsules (**Figure 1E**). These results indicate that the higher lignin deposition in the capsule cell walls of BG than those of SG is solely responsible for the thicker pericarps of BG than SG.

### Identification of pericarp thickness-related metabolites in BG and SG

**Figure 2:**
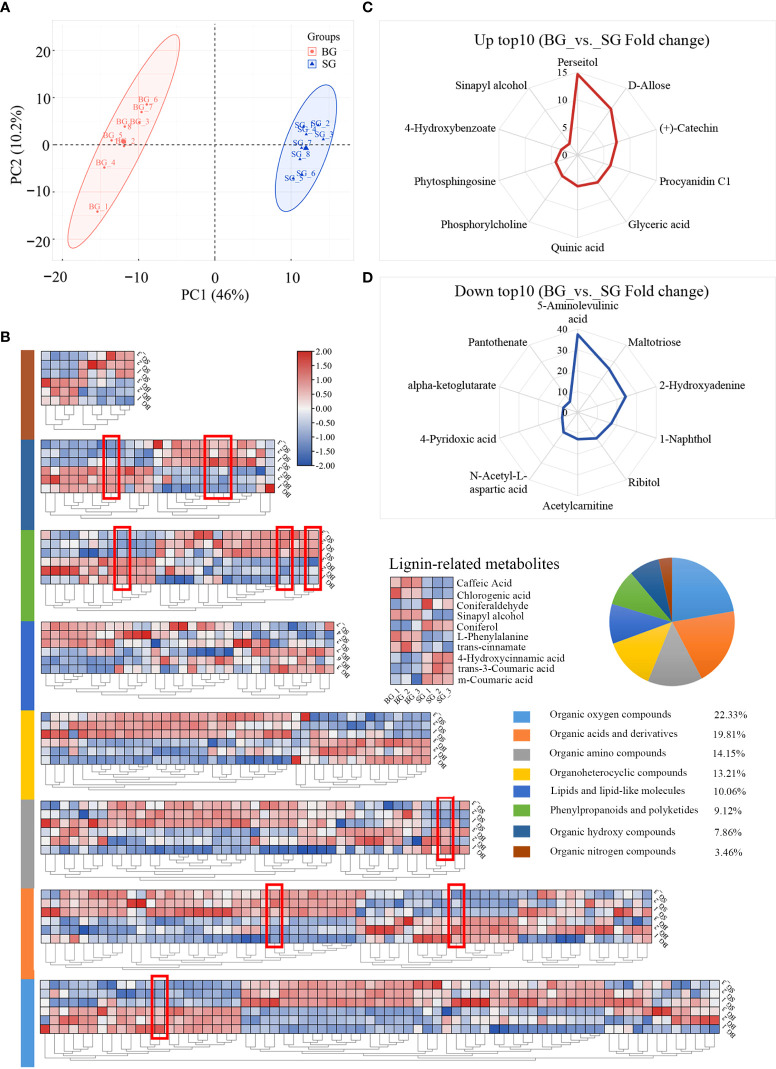
Component analysis of the identified metabolites based on UHPLC-Q-TOF MS. **(A)** Principal component analysis (PCA) showing all samples. **(B)** Heat map of the identified metabolite expression levels and pie chart showing the superclass of these metabolites between BG and SG. **(C)** Top 10 metabolites with upregulated expression in BG vs. SG. **(D)** Top 10 metabolites with upregulated expression in SG vs. BG.

We analyzed the metabolite profiles of the BG and SG capsules and determined their correlation with pericarp thickness. Nontargeted UHPLC-Q-TOF MS-based metabolite profiling in positive and negative ion modes revealed 318 metabolites. These metabolites were classified into eight groups and included eight major compounds in the monolignol biosynthetic pathway (**Figure 2B** and **Supplementary Table S2**).

The first component of the PCA results (46%) predominantly reflected the difference between SG and BG, and the second component (10.2%) primarily indicated within-group differences, which separated the BG and SG samples in terms of metabolite composition and content (**Figure 2A**). The differentially accumulated metabolites (DAMs) were represented based on variable importance in the projection score (VIP > 1.0), fold change (FC > 2.0 or FC < 0.5), and p-value (p < 0.05. To eliminate the effects of quantity on pattern recognition, we applied Z-score transformation of the peak areas for each metabolite and subsequently performed hierarchical cluster analysis. As shown in **Figures 2B–D**, sinapyl alcohol (S-unit, one generic primary monolignol) and organic oxygen compounds were the most upregulated metabolites while organic acids and derivatives were the most downregulated metabolites in the BG capsules. Despite the fact that coniferyl alcohol (G-unit) and coumarate conjugate (H-unit) were also viewed as essential monomers of lignin, the former (G-unit) showed no significant difference in amount between the BG and SG capsules, and the latter (H-unit) was undetectable in both species (**Supplementary Table S2**). Sugar units, as typical monomers of cellulose and hemicellulose, were at similar levels in the two species, suggesting that the accumulation of S-lignin was the key factor contributing to the distinct pericarp thickness of the two species.

### Transcriptome profiles of BG and SG and their phylogenetic relationship

**Figure 3:**
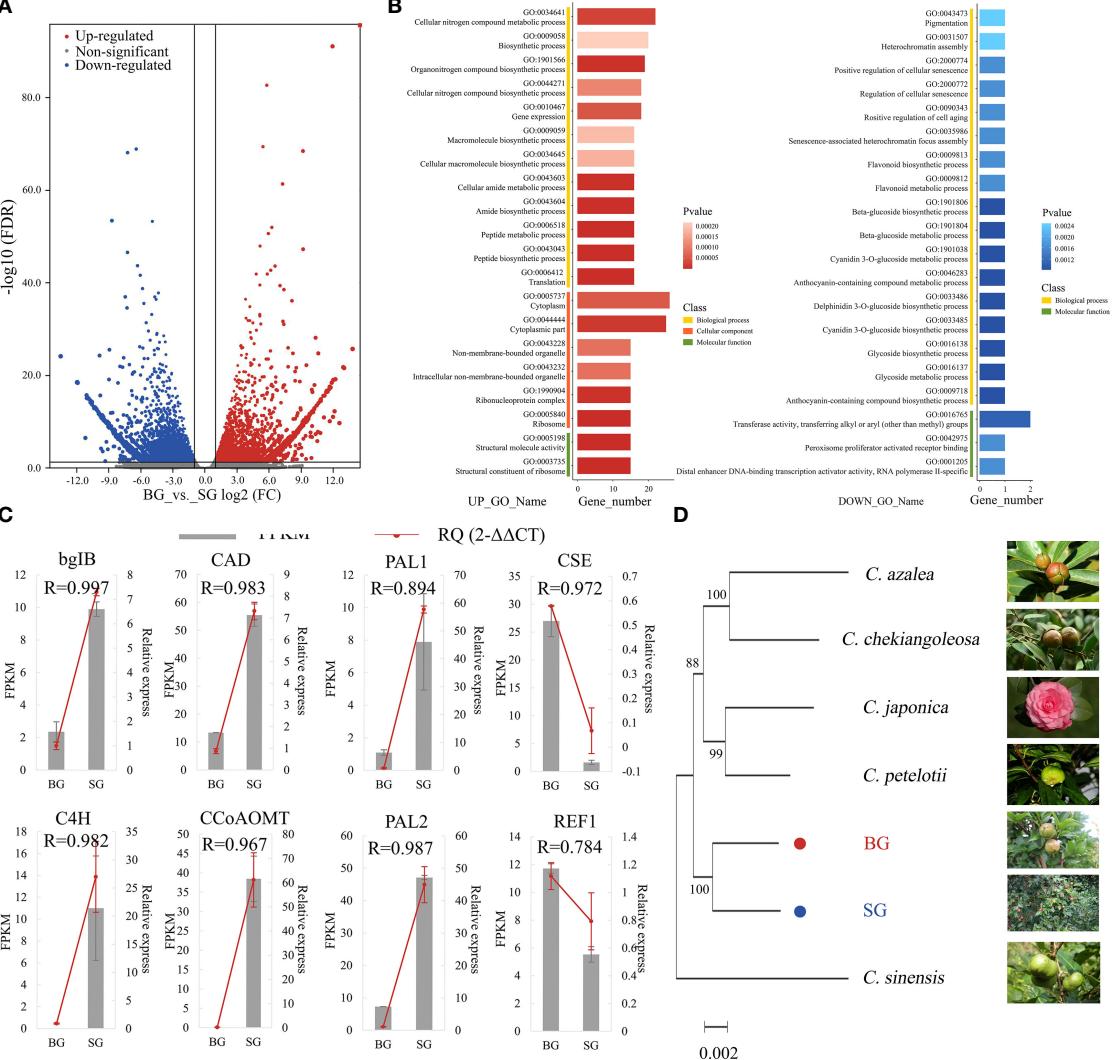
Transcriptome data comparison between BG and SG. **(A)** Volcano plot analysis of differentially expressed genes (DEGs), |log2(FC)|>1; **(B)** Gene Ontology (GO) functional annotation terms (UP_TOP 20 and DOWN_TOP 20) **(C)** Comparison of the expression levels of eight lignin genes in FPKM and RT-PCR. Error bars represent SE. **(D)** Molecular phylogenetic analysis by the maximum likelihood method of single-gene orthologs in seven Camellia species.

The transcriptome profiles of BG and SG were obtained and compared to investigate the transcriptional regulation of DAMs in pericarps from the BG and SG samples. Approximately 245.07 Gb of raw data was generated, and the statistics of the sequencing libraries are summarized in **Table 1**. Unique-mapped reads were used to calculate the expression levels in transcripts per million (Wagner et al., 2012; Vera Alvarez et al., 2018). The resulting sets yielded 2.5 × 10^8^ clean reads, with over 60% mapped to the assembly transcripts from RNA-seq data in non-model organisms (**Table 1**). A total of 337,768 protein-coding genes were predicted in NR, Swissprot, PFAM, GO, and KO, of which 8974 DEGs; 4322 upregulated and 4652 downregulated) were identified with the criteria of a false discovery rate (FDR) < 0.05 and a |log2FC | > 1 (**Figure 3A** and **Supplementary Table S3**).

**Table 1:** Data quality of each sample in BG and SG.

Sample	Raw_reads	Clean_reads	Clean_bases	Error (%)	Q20 (%)	Q30 (%)	GC (%)	Total mapped
BG_1	44887502	44561462	6.18G	0.03	97.73	93.3	44.52	27512858 (61.74%)
BG_2	44739036	44469648	6.18G	0.03	97.79	93.41	44.5	27410784 (61.64%)
BG_3	43875258	43563742	6.05G	0.03	97.77	93.4	44.88	27394846 (62.88%)
SG_1	41097020	40819936	5.67G	0.03	97.72	93.29	44.69	25812610 (63.24%)
SG_2	41678902	41427112	5.75G	0.03	97.75	93.33	44.29	26452142 (63.85%)
SG_3	40699122	40422478	5.61G	0.03	97.75	93.35	44.2	25892220 (64.05%)

GO functional enrichment analyses of DEGs were assigned to biological processes (72.2%), molecular functions (87.0%), and cellular components (64.8%). Among the biological processes, metabolic and macromolecular biosynthetic processes were highlighted. Meanwhile, most of the DEGs were enriched in “structural molecule activity” and “structural constituent of ribosome” molecular function terms toward structural molecular activity, which is consistent with the DAMs profile (**Figure 3B** and **Supplementary Table S4**). qRT-PCR results validated the RNA-seq results obtained using eight monolignol biosynthesis-related DEGs. The expression patterns of these genes obtained using qRT-PCR were consistent with those determined by RNA-seq (**Figure 3C**). For the nuclear *Camellia* phylogeny, we used transcriptomic sequences of BG and SG and combined them with public transcriptome datasets from six other *Camellia* species to identify 334 conserved single-copy nuclear genes (**Supplementary Table S5**). The smallest gene set was then subjected to phylogenetic analysis using the maximum-likelihood method, which highly supported a divergence of BG and SG after the most recent common ancestor of *C. oleifera*, suggesting the feasibility of frequent interspecific hybridization and genetic introgression between the two species during the evolution and domestication of *Camellia* (**Figure 3D**).

### Identification of novel transcription factors that regulate lignin metabolism in *Camellia*

**Figure 4:**
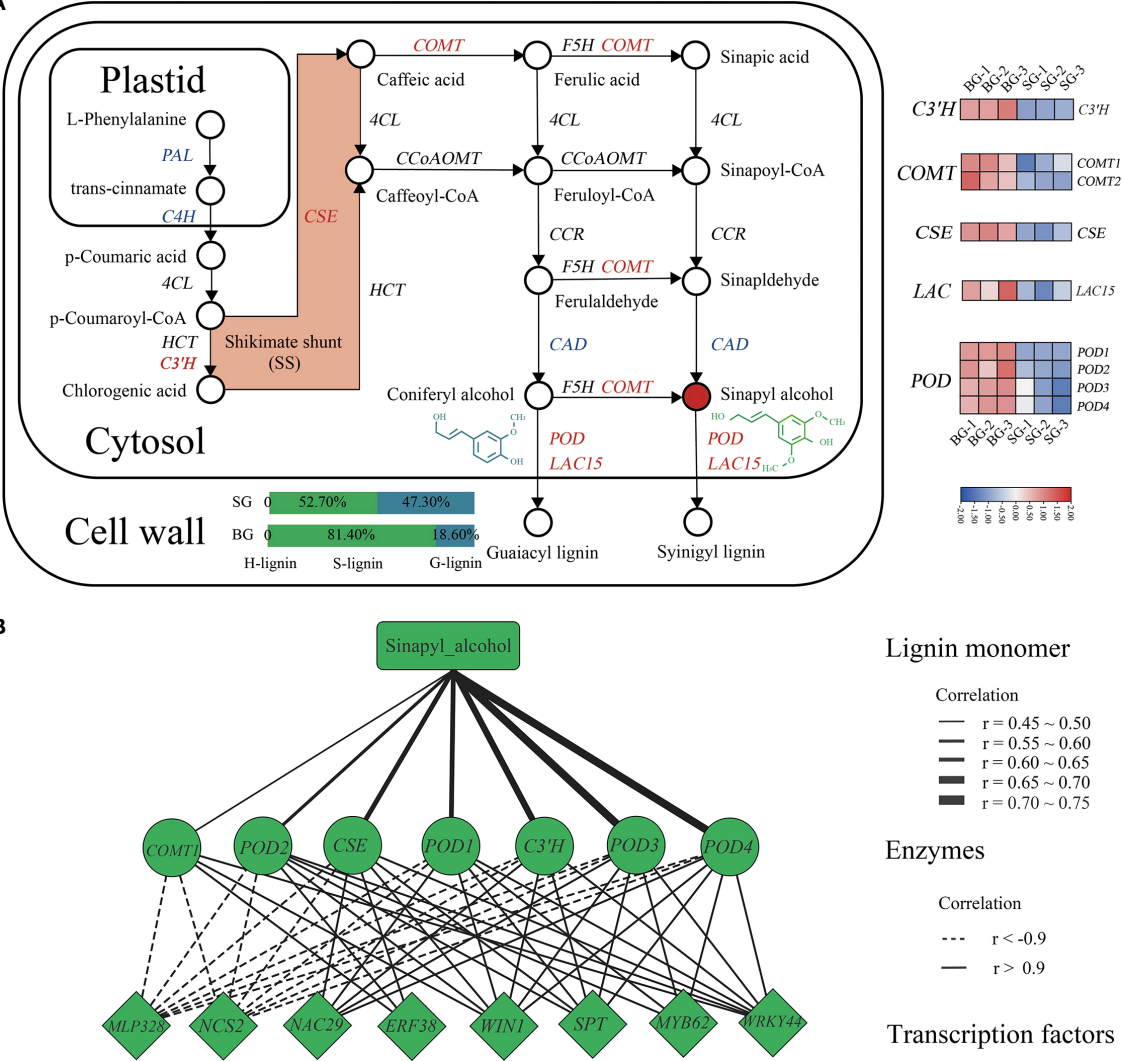
Analysis of the lignin biosynthetic pathways of BG and SG capsules. **(A)** Map showing the lignin biosynthetic pathway for patterns of DEGs. Expression of lignin-related genes is shown by a heatmap using log10 (FPKM); red represents upregulation, and blue represents downregulation. **(B)** Coexpression network of lignin biosynthesis end products, upregulated DEGs, and transcription factors.

To provide an overview of the DAMs and DEGs involved in the lignin biosynthetic pathway, we mapped the metabolic compounds and catalytic enzymes onto the pathway based on the KEGG database. A moderate proportion of the genes and compounds involved in the lignin biosynthetic pathway, including precursors, intermediates, end products, and sequential enzymes, were distinct between the SG and BG capsules (**Figure 4A**). Based on the KEGG database, we identified 11 catalytic enzymes in *Camellia* that are potentially involved in the biosynthetic pathway to the major monolignol precursors of lignin. The expression levels of genes encoding p-coumarate 3-hydroxylase (C3’H), caffeoyl shikimate esterase (CSE), and caffeic acid *O*-methyltransferase (COMT) were increased in BG, leading to a predominance of S-units (81.4%), accompanied by low levels of G-units (18.6%) despite reduced expression levels of cinnamyl alcohol dehydrogenase (*CAD*), cinnamate 4-hydroxylase (*C4H)*, and L-phenylalanine ammonia-lyase (*PAL)*. The oxidative polymerization of monolignols is catalyzed by peroxidases (PODs, using hydrogen peroxide) and laccases (LACs, using molecular oxygen) (Liu et al., 2018; Dixon and Barros, 2019). We also identified four *POD* genes and one *LAC* gene in *Camellia* that were homologous to *AtPRX* and *AtLAC*15 in *Arabidopsis*, which might enhance monolignol bulk polymerization in BG through their upregulated expression (**Figure 4A** and **Supplementary Table S6**).

To further explore the transcription factors (TFs) regulating lignin metabolism in *Camellia*, we performed a coexpression network analysis to direct differentially expressed TFs toward genes encoding S-lignin closely related enzymes (i.e., COMT1, POD2, CSE, POD1, C3’H, POD3, and POD4), and then narrowed down eight hub TFs into a highly correlated key module (|correlation| > 0.9) (**Figure 4B**). Among these TFs, wax inducer1/SHINE1 (WIN1/SHN) and ethylene response factor 38 (ERF38), which belong to the AP2/ERF family, were positively correlated with lignin metabolism in *Camellia*, as did MYB62, NAC29, SPATULA (SPT), and WRKY transcription factor 44 (WRKY44), whereas MLP328 and NCS2, which belong to the pathogenesis-related (PR) protein family, were negatively correlated with all enzymes previously mentioned (**Figure 4B** and **Supplementary Table 6**). Taken together, the results indicated that these TFs, based on their annotated orthologs in *Arabidopsis*, were hypothesized to be the master regulators (activators and/or repressors) of pericarp lignification in *Camellia*.

## Discussion

*Camellia* L. is a good oil feedstock because of the biomass accumulation ability of its seeds embedded in lignocellulosic pericarps; therefore, pericarps with thin and low-density lignocellulose are favorable because they make seeds accessible (Yan, 2020). The pericarp thickness variations between BG and SG primarily arise from their genetic distinction, providing insights into the growth–defense tradeoffs in *Camellia* against stresses during evolution and domestication.

### High S-lignin is the key factor contributing to the thicker fruit pericarp of BG compared with SG

Mature *Camellia* seeds are generally protected by a rigid pericarp (approximately 1.2–7.0 mm) against external hazards, and pericarp thickness is mainly determined by secondary cell wall components, such as cellulose, hemicellulose, lignin, and a small proportion of other components (Tan et al., 2020; **Figure 1**). The cellulose and hemicellulose contents of the BG and SG capsules were nearly the same, but the lignin content of the BG pericarp was higher than that of the SG pericarp, implying that lignin accumulation was responsible for the thick pericarp of *Camellia* (**Figure 1E** and **Supplementary Table S8**).

Lignin is the second most abundant biopolymer that polymerizes on the cell wall surface (Ralph et al., 2004). The three essential monolignols are p-hydroxyphenyl (H, derived from 4-coumaryl alcohol), guaiacyl (G, derived from coniferyl alcohol), and syringyl (S, derived from sinapyl alcohol) units (Boerjan et al., 2003; Bonawitz and Chapple, 2010; Liu et al., 2018). Comparison of metabolic profiles showed that S-unit was the only differentially accumulated monolignol among the three main monolignols. Thus, the S/G ratio was higher in the BG capsules than in the SG capsules. This phenomenon was accompanied by the attenuation of flavonoid biosynthesis *via* the shikimate pathway and consequently increased synthesis of monolignols from shikimate and phenylalanine (Vogt, 2010; **Figures 2B**, **4** and **Supplementary Table S2**).

### Regulation of several catalysts and TFs in the lignin biosynthetic pathway contributes to high accumulation of S-lignin in BG capsules

In this study, we demonstrated the biosynthetic pathway to the major monolignol precursors of lignin in *Camellia*: from phenylalanine *via* the phenylpropanoid pathway and subsequent monolignol polymerization *via* PODs and LACs (**Figure 4A**). The catalytic enzymes, as in most plants, comprise the deaminase PAL, which converts phenylalanine to cinnamic acid, and hydroxylases, methyl/acyl-transferases, reductases, and oxidases for polymerization (Liu et al., 2018; Dixon and Barros, 2019). We speculated that the high level of S-unit in the BG capsules was associated with activated shikimate shunt, which was catalyzed by the increased activities of C3’H and CSE, supplying an abundant shikimate pool despite the decreased activities of CADs (A. Wagner et al., 2007; **Figure 4**). Finally, oxidative polymerization ultimately determined the output of S-lignin, which was also likely boosted in the BG capsules for the increased activities of PODs and LACs (**Figure 4A**).

The most widely studied TFs in the regulation of lignin biosynthesis are those belonging to the MYB and NAC families, including MYB58 and MYB63 in *Arabidopsis*, MYB31 in *Musa*, SND1 and NST1 in *Arabidopsis*, and NAC141 in *Eucalyptus*, which act as activators or repressors (Legay et al., 2007; Zhong et al., 2007; Sun et al., 2021). In the present study, MYB62 and NAC29 were screened through the coexpression analysis of the RNA-seq dataset for their potential role in regulating the lignin biosynthetic pathway in *Camellia* (**Figure 4B** and **Supplementary Table S7**). Additionally, the expression of conventional growth-related factors *WRKY*s and *AP2*/*ERFs*, such as AtWRKY12 (Li et al., 2015), PtrWRKY19 (Yang et al., 2016), and *OsSHN*/*WIN* (Ambavaram et al., 2011), has been associated with lignin contents in some plants. Meanwhile, WRKY44 and WIN1 in *Camellia*, which are positively correlated with catalysts in the lignin biosynthetic pathway, might function as activators. *SPT*, which encodes a bHLH TF, was originally identified for its role in carpel and fruit development (Groszmann et al., 2008; Makkena and Lamb, 2013). Thus, it was presumed to divert the S-lignin biosynthetic metabolon specifically into *Camellia* pericarps in the present study. Intriguingly, two defense-induced genes, *pathogenesis-related 2*, were negatively correlated with these catalysts, which could explain the weak defense in BG.

### Alteration in lignin biosynthesis might be a strategy evolved by *Camellia* to balance growth and defense

Plant growth–defense tradeoffs involve resource reallocation to different biological processes, whereby plants optimize performance and fitness in a dynamic environment (Xie et al., 2018). Lignin biosynthesis is commonly believed to be an indivisible part of the complicated crosstalk between growth and defense because lignin provides mechanical strength, transports water and nutrients, and acts as a physical barrier to pathogen ingress; however, the relationship of lignin content with growth and defense remains largely elusive (Ha et al., 2021). Our previous study showed that S-lignin enrichment in BG pericarp co-occurs with increased growth rate and weak immunity, which differ from those in SG pericarp with low lignin content (our unpublished data). Similar findings have been reported in studies of *Arabidopsis* MYB46 and quinate/shikimate p-hydroxy cinnamoyl transferase (HCT) mutants, where reduced lignin content triggers stunt growth and enhances defense (Gallego-Giraldo et al., 2011).

**Figure 5:**
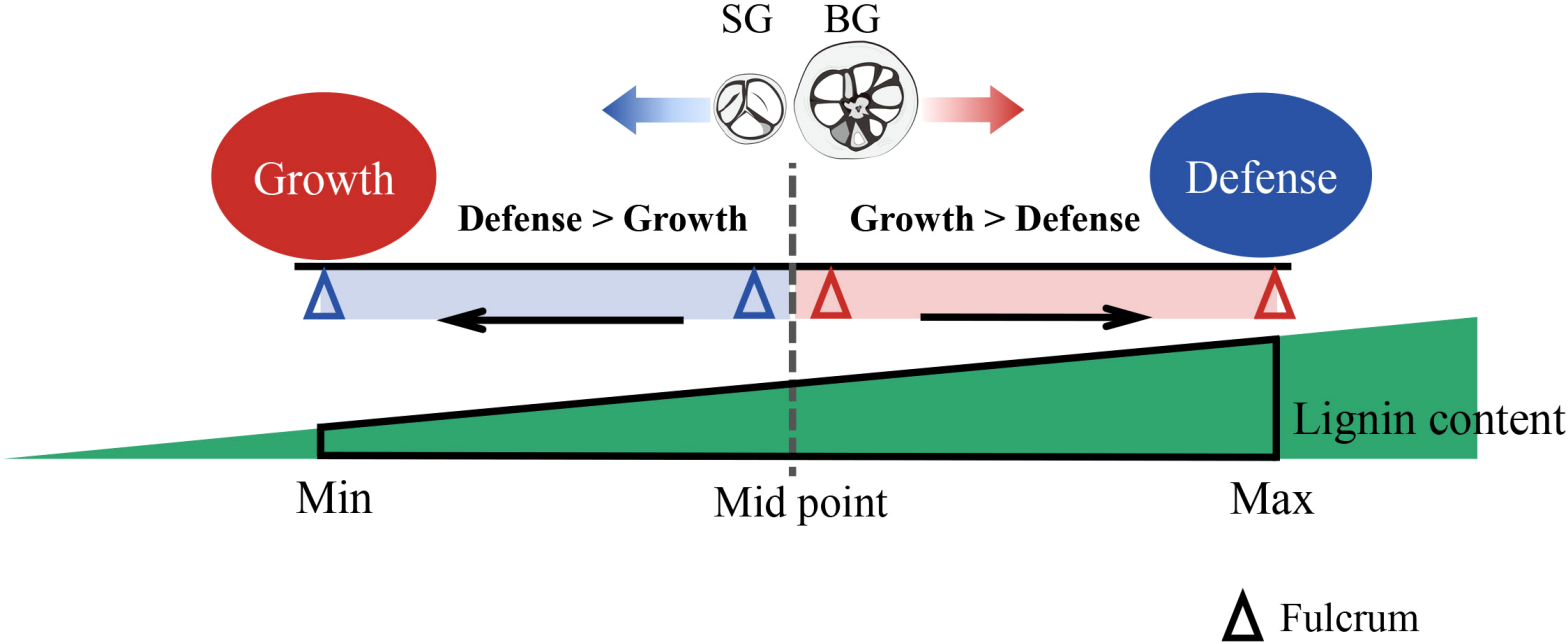
Proposed schematic of lignin-regulated growth–defense tradeoffs in Camellia. Lignin content (red triangle) within a certain threshold (from Min to Max) acts as a fulcrum to balance the resource flux into the plant growth and defense: A tendency of growth bias (BG) or defense bias (SG) is precisely tuned by the lignin level in pericarps. BG, *C. drupifera*; SG, *C. oleifera*; S-lignin, syringyl lignin; GO, gene ontology; KEGG, Kyoto Encyclopedia of Genes and Genomes; TBO, toluidine blue O; OPLS-DA, orthogonal partial least-squares discrimination analysis; VIP, variable importance in projection; DAMs, differentially accumulated metabolites; DEGs, differentially expressed genes; FC, fold change; FDR, false discovery rate; qRT-PCR, quantitative real-time polymerase chain reaction; PCA, principal component analysis; S-unit, sinapyl alcohol; G-unit, coniferyl alcohol; H-unit, coumarate conjugate; C3’H, p-coumarate 3-hydroxylase; CSE,S caffeoyl shikimate esterase; COMT, caffeic acid O-methyltransferase; CAD, cinnamyl alcohol dehydrogenase; C4H, cinnamate 4-hydroxylase; PAL, L-phenylalanine ammonia-lyase; POD, peroxidases; LAC, laccases; TF, transcription factor; ERF38, ethylene response factor 38; WIN1/SHN, wax inducer1/SHINE1; SPT, SPATULA; WRKY44, WRKY transcription factor 44; PR, pathogenesis-related; HCT, quinate/shikimate p-hydroxy cinnamoyl transferase.

During the evolution and domestication of *Camellia*, frequent interspecific hybridization and genetic introgression between *C. drupifera* and *C. oleifera* would be conceivable because of their phylogenetically close relationship (**Figure 3D**). Thus, lignin content variation became prominent and was associated with the evolutionary adaptation of *Camellia* species. The large amount of energy invested in lignin accumulation can compensate for the costly expenditure of defense, producing a growth-bias phenotype. However, excessive lignin accumulation also inhibits growth. Thus, within a certain threshold, lignin content possibly acts as a “fulcrum” that balances the flux of resources, such as carbon and energy, into growth and defense, helping *Camellia* species regulate their response to environmental changes (**Figure 5**). Whether or not the model we established about the lignin-dependent growth–defense tradeoffs in SG and BG can be extended to other *Camellia* species requires validation but could serve as a basis for further exploration.

## Data availability statement

The original contributions presented in the study are publicly available. This data can be found here: NCBI, PRJNA870661.

## Author contributions

Data curation, investigation, visualization, writing–original and draft, YJL; Conceptualization, funding acquisition, project administration, writing–review and editing, BL; Formal analysis and writing–original draft, YW; Data curation, HL; Investigation, KZ, BY, and SL; Visualization, WS; Methodology, CL and WS; Conceptualization, supervision, writing-review, and editing, BZ; Funding acquisition, formal analysis and project administration, YQL. All authors have read, corrected, and approved the manuscript.

## Funding

This research was funded by the Key-Area Research and Development Program of Guangdong Province, grant number 2020B020215003 and the Guangzhou Municipal Science and Technology Project, grant number 202201011754.

## Acknowledgments

We thank Xiaokeng Forest Farm (Shaoguan City, Guangdong Province, China) and Boluo Forest Farm (Huizhou City, Guangdong Province, China) for providing plant materials. We thank Shanghai Applied Protein Technology Co. Ltd. For assisting us with the metabolic and transcriptome data analyses. We thank Bioruqi Technology Co., Ltd. (Guangzhou, China) for assisting us with qPCR experiments and Hunan Platte Network Technology Co., Ltd. For assisting us with toluidine blue reagent staining experiments.

## Conflict of interest

The authors declare that the research was conducted in the absence of any commercial or financial relationships that could be construed as a potential conflict of interest.

## Publisher’s note

All claims expressed in this article are solely those of the authors and do not necessarily represent those of their affiliated organizations, or those of the publisher, the editors and the reviewers. Any product that may be evaluated in this article, or claim that may be made by its manufacturer, is not guaranteed or endorsed by the publisher.

## Supplementary material

Supplementary Table 1 Result of RT-PCR.

Supplementary Table 2 Analysis of the identified metabolite profiles between BG and SG.

Supplementary Table 3 DEGs obtained after comparing the transcriptome data of BG and SG.

Supplementary Table 4 GO functional enrichment analysis performed on DEGs.

Supplementary Table 5 334 conserved single-copy nuclear genes.

Supplementary Table 6 Identified PODs and LACs in Camellia.

Supplementary Table 7 End product, upregulated DEGs, and 10 hub TFs of the coexpression net-work in lignin biosynthesis.

Supplementary Table 8 Comparison of fruit phenotypic character-istics between two species of Camellia L.
